# Effects of Tasimelteon Treatment on Traumatic Brain Injury Through NRF-2/HO-1 and RIPK1/RIPK3/MLKL Pathways in Rats

**DOI:** 10.1007/s12035-025-04711-0

**Published:** 2025-01-29

**Authors:** Eyyüp Sabri Özden, Mustafa Soner Özcan, Mehtap Savran, Ilter Ilhan, Muhammet Yusuf Tepebası, Mehmet Abdulkadir Sevuk, Özlem Özmen

**Affiliations:** 1 Department of Anesthesiology and Reanimation, Faculty of Medicine, Suleyman Demirel University, Cunur, 32260 Isparta, Turkey; 2 Department of Pharmacology, Faculty of Medicine, Suleyman Demirel University, Isparta, Turkey; 3 Department of Biochemistry, Faculty of Medicine, Suleyman Demirel University, Isparta, Turkey; 4 Department of Genetic, Faculty of Medicine, Suleyman Demirel University, Isparta, Turkey; 5 Department of Pathology, Faculty of Veterinary Medicine, Burdur Mehmet Akif Ersoy University, Burdur, Turkey

**Keywords:** Traumatic brain injury, Tasimelteon, NRF-2/HO-1, RIPK1/RIPK3/MLKL, Neuroprotection

## Abstract

Secondary brain damageafter traumatic brain injury (TBI) involves oxidative stress, neuroinflammation, apoptosis, and necroptosis and can be reversed by understanding these molecular pathways. The objective of this study was to examine the impact of tasimelteon (Tasi) administration on brain injury through the nuclear factor erythroid 2-related factor 2 (NRF-2)/heme oxygenase-1 (HO-1) and receptor-interacting protein kinase 1 (RIPK1)/receptor-interacting protein kinase 3 (RIPK3)/mixed lineage kinase domain-like (MLKL) pathways in rats with TBI. Thirty-two male Wistar albino rats weighing 300–350 g were randomly divided into four groups: the control group, trauma group, Tasi-1 group (trauma + 1 mg/kg Tasi intraperitoneally), and Tasi-10 group (trauma + 10 mg/kg Tasi intraperitoneally). At the end of the experimental phase, after sacrifice, blood samples and brain tissue were collected for biochemical, histopathological, immunohistochemical, and genetic analyses. Tasi increased the total antioxidant status and decreased the total oxidant status and oxidative stress index. In addition, Tasi caused histopathological changes characterized by a markedly reduced hemorrhage area in the Tasi-1 group. Normal brain and meningeal structure was observed in rats in the Tasi-10 group. Immunohistochemical analysis indicated that Tasi also decreased the expression of interferon-gamma, caspase-3, and tumor necrosis factor-alpha in the brain tissue. Although NRF-2 and HO-1 expression decreased, RIPK1/RIPK3/MLKL gene expression increased due to trauma. However, Tasi treatment reversed all these findings. Tasi protected against brain injury through the NRF-2/HO-1 and RIPK1/RIPK3/MLKL pathways in rats with TBI.

## Introduction

Traumatic brain injuries (TBIs) are typically the result of traumatic incidents, such as accidents, falls, or violent acts. These injuries often result in structural or functional damage to brain tissue. The underlying molecular mechanisms of TBI are initiated by the interaction of neurotransmitters, biochemical mediators, cytokines, and genetic alterations. Excitotoxicity, including the overactivation of neurotransmitters, disturbances in calcium homeostasis, nitric oxide production, and reactive oxygen species (ROS) production, which contribute to cell death through apoptosis, represents a pathophysiological factor in this event. These molecular events can trigger apoptotic pathways that exacerbate tissue damage. Additionally, injured brain tissue can trigger an inflammatory response that can both impair and facilitate repair processes through immune activation [1].

Following TBI, an exaggerated immune response has the potential to damage the central nervous system (CNS), ultimately leading to neurological impairment. Inflammatory substances such as tumor necrosis factor-alpha (TNF-α) and interferon-gamma (IFN-γ) are secreted in response to the activation of microglia and astrocytes. The overproduction of proinflammatory cytokines can exacerbate neuronal and axonal damage, disrupt the blood–brain barrier (BBB), and increase intracranial pressure. The inhibition of inflammatory pathways has been shown to reduce cytokine levels and affect apoptosis-related proteins, thus providing neuroprotective and therapeutic benefits. The primary forms of cell death after TBI are apoptosis and necrosis. This type of cell death is initiated by an imbalance in proapoptotic and antiapoptotic proteins through the caspase-3 (Cas-3) pathway [1].

Chronic microglial activation has been shown to increase the production of cytotoxic mediators, including TNF-α, high mobility group Box 1, and inducible nitric oxide synthase, which in turn leads to the accumulation of ROS. Neuroinflammation can be mitigated by activating the nuclear factor erythroid 2-related factor 2 (NRF-2) pathway, which results in the induction of various antioxidant enzymes that help reduce oxidative damage and neuronal loss. The transcription factor NRF-2 plays a crucial role in maintaining redox balance within the body and provides neuroprotection against oxidative stress in vivo [1].

Heme oxygenase-1 (HO-1), a crucial component of the cellular defense system, is activated in response to oxidant-induced damage. In the CNS, HO-1 has been demonstrated to possess antinecroptotic, antineuroinflammatory, and neuroprotective functions [2].

Necroptosis, recently defined as a modulated necrosis pathway and caspase-independent programmed cell death, plays an important role in the pathogenesis of various CNS disorders, including TBI, intracerebral hemorrhage, ischemic stroke, Alzheimer’s disease, Parkinson’s disease, and amyotrophic lateral sclerosis. The activation of receptor-interacting protein kinase-1 (RIPK1) and a TNF ligand family member such as mixed lineage kinase domain-like (MLKL) induces the RIPK1/receptor-interacting protein kinase 3 (RIPK3)/MLKL complex, thereby contributing to necroptosis, a common mechanism of TBI in early brain injury [3].

Melatonin (Mel), the primary secretory product of the pineal gland, plays a pivotal role in immune function, autophagy, anti-inflammatory, antiapoptotic, and antioxidant activities [4]. Tasimelteon (Tasi) is a melatonin receptor agonist that shows promise in reducing secondary brain injury after TBI. Through its action on MT1 and MT2 receptors, Tasi may attenuate inflammatory and oxidative responses by regulating the circadian rhythm. Tasi may protect the blood–brain barrier and limit brain damage by reducing the production of proinflammatory cytokines such as TNF-α and IFN-γ. In addition, Tasi may also promote the production of antioxidant enzymes by activating the NRF-2 pathway and inhibit cell death by increasing HO-1 expression. Owing to these properties, Tasi stands out as a potential neuroprotective agent in TBI treatment [5]. The objective of this study was to examine the impact of Tasi administration on brain injury through the NRF-2/HO-1 and RIPK1/RIPK3/MLKL pathways in rats with TBI.

## Materials and Methods

### Animals

Thirty-two adult male Wistar albino rats weighing 300–350 g were housed in standard Euro-type 4 cages, with each group separated from the others. The rats were housed at 23 °C and 55% humidity with a 12-h light/12-h dark cycle. They were fed ad libitum with standard commercial feed and water. The four experimental groups were formed as follows:

- Control group: Rats were administered 0.5–1 ml of saline (SF) intraperitoneally (IP) without trauma. After 24 h, the rats were sacrificed under anesthesia, and brain tissues were collected.
- In the trauma group, the rats were administered 0.5–1 ml of SF IP for 30 min under anesthesia after trauma was induced. After 24 h, the rats were sacrificed under anesthesia, and brain tissues were collected.
- In the trauma + Tasi 1 mg/kg (Tasi-1) group, the rats were administered 1 mg/kg Tasi IP under anesthesia 30 min after trauma was induced [6]. After 24 h, the rats were sacrificed under anesthesia, and brain tissues were collected.
- In the trauma + Tasi 10 mg/kg (Tasi-10) group, the rats were traumatized under anesthesia. After 30 min, 10 mg/kg Tasi was administered IP. After 24 h, the rats were sacrificed under anesthesia, and brain tissues were collected.

Head trauma was applied by dropping a 50 g ball from a height of 80 cm [7].

The rats were anesthetized with 10 mg/kg xylazine (Xylazin Bio 2%, Bioveta, Czech Republic) + 90 mg/kg ketamine (Keta-Control, Doğa İlaç, Turkey) for the head injury model and were sacrificed. For the sacrificial procedure, surgical exsanguination was performed by drawing blood from the inferior vena cava. After sacrifice, blood samples and brain tissue were collected. Half of the tissues were placed in formaldehyde solution for histopathological examination. The other halves of the tissues were placed in Eppendorf tubes and stored at − 80 °C for biochemical and genetic analyses. Biochemical indicators of oxidative stress (total antioxidant status (TAS), total oxidant status (TOS), and the oxidative stress index (OSI)) were analyzed in blood samples and brain tissues. The brain tissues were stained with hematoxylin and eosin (HE) for histopathological examination. The levels of pathological findings such as edema, congestion, hemorrhage, and necrosis were determined, and IFN-γ, Cas-3, and TNF-α levels were analyzed via immunohistochemical staining. In addition, the mRNA expression levels of genes related to oxidative stress and necrosis-related pathways (NRF-2/HO-1 and RIPK1/RIPK3/MLKL) were analyzed.

### Biochemical Analysis

To initiate the experiment, the tissues were diluted in phosphate-buffered saline (10 mM sodium phosphate) with a tenfold weight/volume ratio, and the pH was adjusted to 7.4. The tissues were subsequently homogenized via a tissue homogenizer (IKA Ultra Turrax T25, Janke & Kunkel, Staufen, Germany). Following homogenization, the samples were centrifuged at 10,000 rpm for 100 min at + 4 °C via a Nuve NF 1200R centrifuge (Ankara, Türkiye). The supernatant obtained after centrifugation was utilized to measure the concentrations of tissue TAS and TOS. An automated biochemistry analyzer (Beckman Coulter AU 5800, Brea, CA, USA) and colorimetric methods were employed for these assays [8, 9]. The TOS results are expressed as µmol H2O2 eq./l, whereas the TAS results are reported as mmol Trolox eq./l. The OSI was calculated by dividing TOS levels by TAS levels, denoted as OSI = TOS/TAS × 100 [10].

### Histopathological Method

At the conclusion of the experimental phase of the study, all the rats in each group were euthanized. To preserve the integrity of the brain tissue during necropsy, the brains of the rats were carefully removed from the skull after gentle dissection. The brain samples from each group were then carefully examined grossly. The samples were subsequently fixed in a 10% buffered formalin solution. Normal tissue processing procedures were followed, including embedding the brain tissues in paraffin. Serial 5-µm-thick sections were obtained from each tissue sample via an automated rotary microtome. These sections were stained via routine HE staining techniques and examined under a light microscope.

The criteria listed in Table 1 were employed to evaluate the histopathological data, similar to the approach used in the study by Mielke et al. [11]. The possible scores ranged from 0 to 4.

**Table 1:** The criteria employed to evaluate the histopathological data

**Histopathological scores of subarachnoid hemorrhages**	
**0**	Normal meningeal and parenchymal structure
**1**	No blood in the subarachnoid space, ventricles, or brain parenchyma.
**2**	No localized or diffuse thin subarachnoid hemorrhage, intraventricular, or intraparenchymal hemorrhage.
**3**	No diffuse or localized thick subarachnoid blood layers, intraventricular, or intraparenchymal hemorrhage.
**4**	Intraventricular or intraparenchymal hemorrhage in association with subarachnoid hemorrhage, regardless of thickness or location.

### Immunohistochemical Method

Sections were mounted on poly-L-lysine-coated slides and subjected to immunohistochemical staining via the streptavidin–biotin peroxidase method. Primary antibodies targeting IFN-γ (IFN-γ antibody (37,895) (Novus Biological, Oxon, UK), Cas-3 (anti-caspase-3 antibody [EPR18297] (ab184787) (Abcam, Cambridge, UK), and TNF-α (recombinant anti-TNF-α antibody [EPR21753-109] (ab205587) (Abcam, Cambridge, UK) were used for the immunohistochemical analysis of brain sections. All primary antibodies were diluted at a ratio of 1/100 using antibody dilution solutions. The immunohistochemistry process was conducted according to the manufacturer’s instructions. The Mouse and Rabbit Specific HRP/DAB Detection Kit-Micropolymer (ab236466) (Abcam, Cambridge, UK) was utilized as the secondary kit in this investigation. While other steps were performed as directed, for negative controls, antibody dilution solutions were applied to the sections at the primary antibody stage instead of primary antibodies.

The percentage of positive cells was quantified via immunohistochemical analysis, and the results were compared and evaluated across the groups. Special attention was given to examining cells from both the control groups and the brain regions affected by trauma. For this purpose, 20 randomly selected cells from each of five areas within the same brain region were counted for each rat via a 40X objective, resulting in a total of 100 cells per area. The number of cells exhibiting a positive immunohistochemical reaction was determined via ImageJ 1.46r software (National Institutes of Health, Bethesda, MD). The results were captured via an Olympus CX41 microscope, and microphotography was performed via the Database Manual Cell Sens Life Science Imaging Software System (Olympus Corporation, Tokyo, Japan).

### Reverse Transcription–Polymerase Chain Reaction (RT–qPCR)

Total RNA was obtained via an RNA isolation kit (Nepenthe, Turkey). The purity and quality of the RNA were measured with a NanoDrop system (Shimadzu Ltd. Kyoto, Japan). cDNA was synthesized from 1 µg of RNA (Atlas Biotechnology, Turkey). Specific mRNA primer sequences were determined via the NCBI website (Table 2). Gene expression levels were determined via real-time PCR (Bio-Rad CFX Connect, CA, USA) with 2X SYBR green master mix (Nepenthe, Turkey). The reaction mixture was prepared according to the manufacturer’s instructions. For normalization, the Rn18s gene was used as a housekeeping gene. Relative mRNA levels were calculated via the 2^−ΔΔCt^ method.

**Table 2:** Primary sequences, product size, and accession numbers of genes

Genes	Primary sequence	Product size	Accession number
**Rn18s (HouseKeeping)**	F: CTCTAGATAACCTCGGGCCG	209 bp	NR_046237.2
	R: GTCGGGAGTGGGTAATTTGC		
**NRF-2**	F: GCCTTCCTCTGCTGCCATTAGTC	126 bp	NM_001399173.1
	R: TCATTGAACTCCACCGTGCCTTC		
**HO-1**	F: AGCCTGGTTCAAGATACTACCTC	240 bp	XM_039097470.1
	R: AGGCCCAAGAAAAGAGAGCC		
**RIPK1**	F: ACCTTAGACGCGTAGGAGCG	527 bp	NM_001107350.1
	R: TCATTGTACTCAGCGCGGTT		
**RIPK3**	F: TAGTTTATGAAATGCTGGACCGC	145 bp	NM_139342.2
	R: GCCAAGGTGTCAGATGATGTCC		
**MLKL**	F: TCCTGGAACTCGGGGTATGG	401 bp	NM_001401077.1
	R: GTTGGCTGACCTCGGAATCA		

*F* forward, *R* reverse, *Rn18s* 18S ribosomal RNA, *NRF-2* nuclear factor E2-related factor 2, *HO-1* heme oxygenase 1, *RIPK1* receptor-interacting protein kinase 1, *RIPK3* receptor-interacting protein kinase 3, *MLKL* mixed lineage kinase domain-like pseudokinase

### Statistical Analysis

For the comparisons between the groups, one-way ANOVA with Tukey’s post hoc test with the GraphPad Prism program was used for statistical analysis, and *p* < 0.05 was considered significant.

## Results

### Tasi Increased Antioxidant Status and Decreased Oxidant Status

**Fig. 1:**
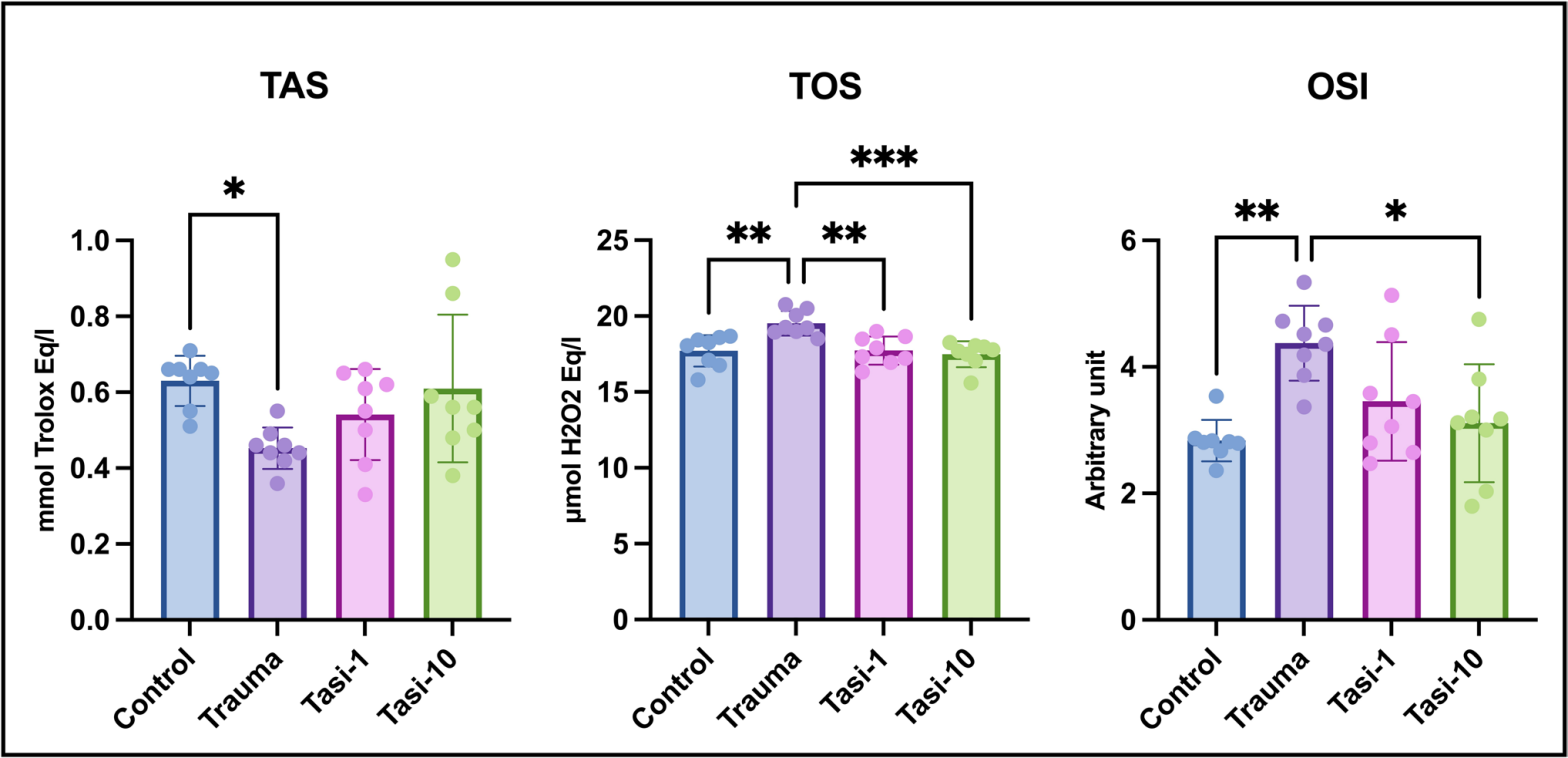
Oxidative stress parameters of brain tissues. Tasi-1, Trauma + Tasi (1 mg/kg); Tasi-10, Trauma + Tasi (10 mg/kg); TAS, total antioxidant status; TOS, total oxidant status; OSI, oxidative stress index. Values are presented as means ± standard deviation. A one-way ANOVA test was used. *p < 0.05, **p ≤ 0.01, ***p ≤ 0.001

Although the TAS values, which are indicators of antioxidant activity, were significantly lower in the trauma group than in the control group (*p* < 0.05), increases in the values of the Tasi-1 and Tasi-10 groups compared with the trauma group were detected, but the differences were not significant. Both the TOS and OSI values, which are indicators of oxidative stress, were significantly greater in the trauma group than in the control group (*p* ≤ 0.01 for both). Meanwhile, the TOS values of the Tasi-1 group were significantly lower than those of the trauma group (*p* ≤ 0.01), and the TOS and OSI values of the Tasi-10 group were also significantly lower than those of the trauma group (*p* ≤ 0.001 and *p* < 0.05, respectively) (Fig. 1).

### Normal Brain Histology in the Tasi-10 Group

**Fig. 2:**
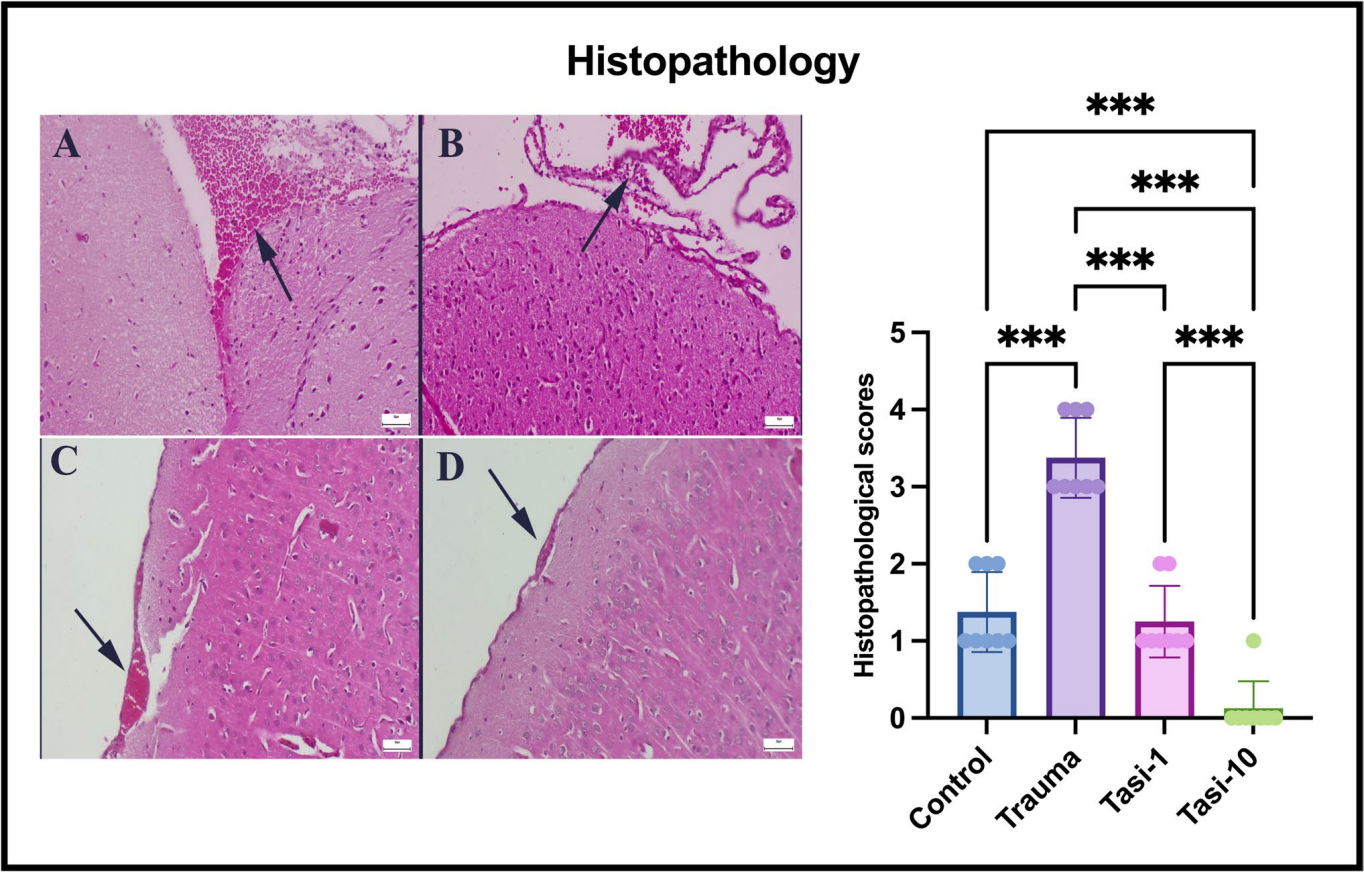
Histopathological appearance and statistical analysis of brains between the groups. **A** Mild hyperemia (arrowhead) and slight hemorrhage (arrow) in meningeal vessels in the control group. **B** Marked hyperemia (arrowhead) and hemorrhage foci (arrow) in the brain of a rat in the trauma group. **C** Markedly reduced hemorrhage area (arrowhead) in the Tasi-1 group. **D** Normal brain and meningeal structure in a rat in the Tasi-10 group, HE, scale bars = 50 µm. Values are presented as means ± standard deviation. A one-way ANOVA test was used. *p < 0.05, **p ≤ 0.01, ***p ≤ 0.001

During the histological examination, mild meningeal hemorrhage was observed in the trauma and Tasi-treated groups. The trauma group of rats presented significant and widespread hemorrhages. Upon treatment with 1 mg of Tasi, the hemorrhagic areas were notably reduced. Normal brain histology was observed in the Tasi-10 group (Fig. 2).

### Tasi Decreased the Expression of IFN-γ, Cas-3, and TNF-α in Brain Tissue

**Fig. 3:**
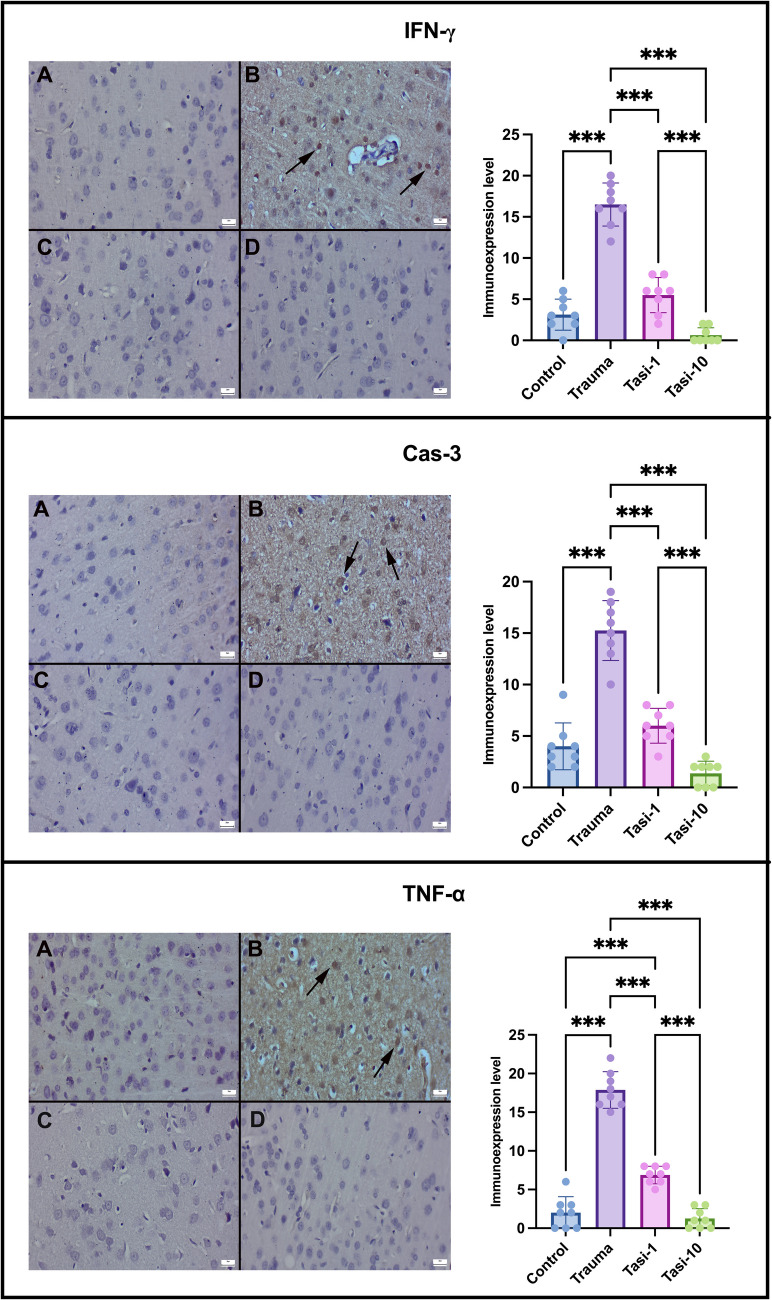
Expression and statistical analysis of IFN-ẟ (top row), Cas-3 (middle row), and TNF-α (bottom row) in the brain. (A) Negative IFN-ẟ, Cas-3, and TNF-α expression in the control group. (B) Significant increase in IFN-ẟ, Cas-3, and TNF-α expressions (arrows) in the trauma group. (C) Marked decrease in IFN-ẟ, Cas-3, and TNF-α expressions in the trauma and Tasi groups. (D) Negative IFN-ẟ, Cas-3, and TNF-α expressions in the Tasi groups, streptavidin–biotin peroxidase method, scale bars = 20 µm. Values are presented as means ± standard deviation. A one-way ANOVA test was used. *p < 0.05, **p ≤ 0.01, ***p ≤ 0.001

The control group exhibited mild to negative expressions of IFN-γ, Cas-3, and TNF-α in their brains following immunohistochemical analysis. The expression of IFN-γ, Cas-3, and TNF-α increased after trauma (*p* ≤ 0.001 for all). The increased expression of all three markers in the trauma group was significantly reduced in the Tasi-1 and Tasi-10 groups (*p* ≤ 0.001 for all). Additionally, the expression levels of all the markers in the Tasi-10 group were significantly lower than those in the Tasi-1 group (*p* ≤ 0.001 for all) (Fig. 3).

### Tasi Increased RIPK1/RIPK3/MLKL Gene Expression and Decreased NRF-2 and HO-1 Gene Expression

**Fig. 4:**
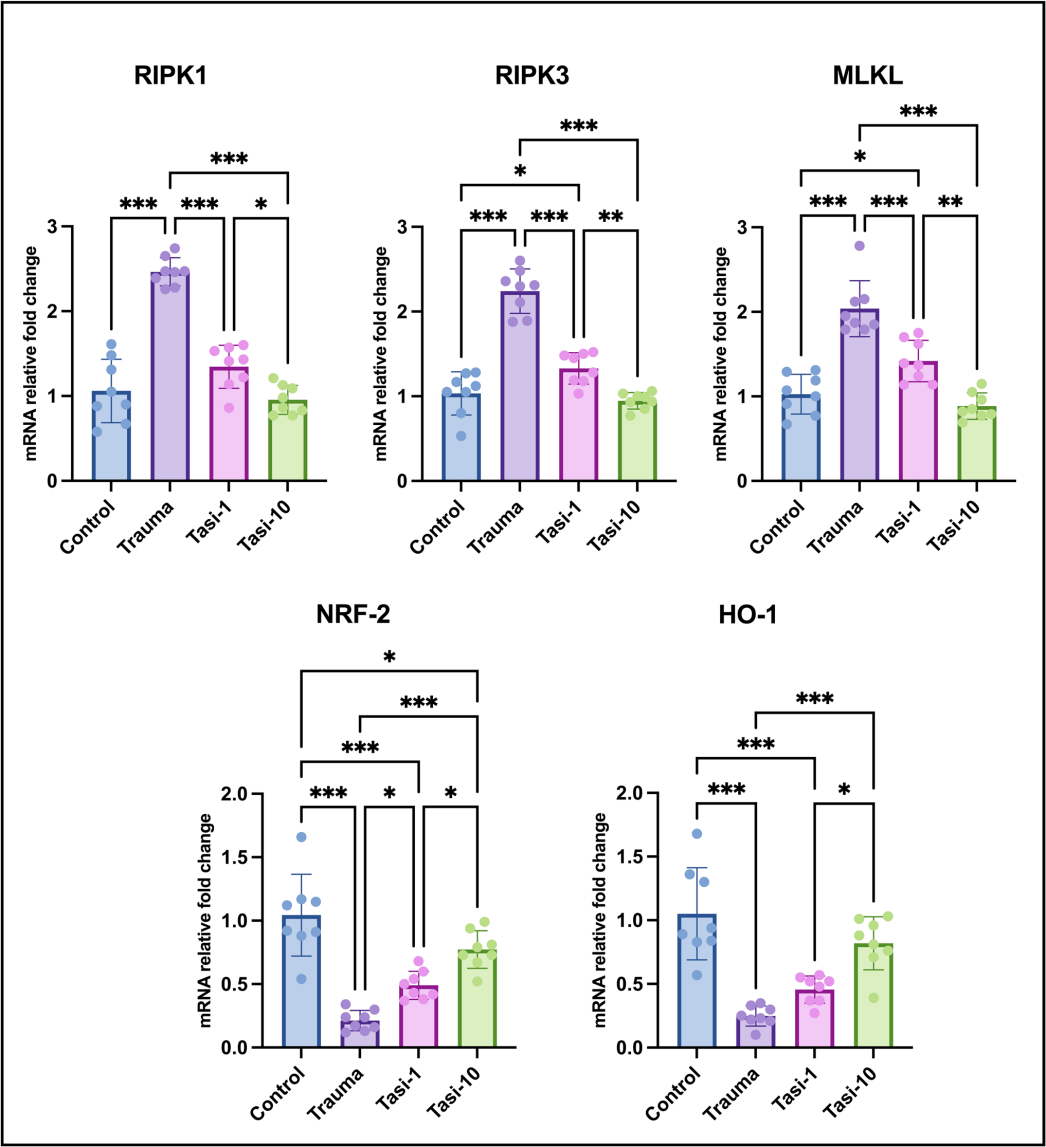
The expression levels of RIPK1, RIPK3, MLKL, NRF-2, and HO-1 in brain tissues. NRF-2, nuclear factor E2-related factor 2; HO-1, heme oxygenase 1; RIPK1, receptor-interacting protein kinase 1; RIPK3, receptor-interacting protein kinase 3; MLKL, mixed lineage kinase domain-like pseudokinase. Values are presented as means ± standard deviation. A one-way ANOVA test was used. *p < 0.05, **p ≤ 0.01, ***p ≤ 0.001

RIPK1/RIPK3/MLKL gene expression was significantly greater in the trauma group than in the control group (*p* ≤ 0.001 for all). RIPK3/MLKL gene expression was significantly greater in the Tasi-1 group than in the control group (*p* < 0.05). In the Tasi-10 group, the expression of all three genes was similar to that in the control group. RIPK1/RIPK3/MLKL gene expression was significantly lower in both Tasi groups than in the trauma group (*p* ≤ 0.001 for all). RIPK1/RIPK3/MLKL gene expression was significantly lower in the Tasi-10 group than in the Tasi-1 group (*p* < 0.05, *p* ≤ 0.01, and *p* ≤ 0.01, respectively). NRF-2 and HO-1 gene expression was significantly lower in the trauma group than in the control group (*p* ≤ 0.001 for both). Meanwhile, NRF-2 and HO-1 gene expression was significantly lower in the Tasi-1 group than in the control group (*p* ≤ 0.001 for both), whereas only NRF-2 gene expression was significantly lower in the Tasi-10 group than in the control group (*p* < 0.05). NRF-2 and HO-1 gene expression was significantly greater in the Tasi-10 group than in the trauma group (*p* ≤ 0.001 for both), whereas only NRF-2 gene expression was significantly greater in the Tasi-1 group than in the trauma group (*p* < 0.05). NRF-2 and HO-1 gene expression was significantly greater in the Tasi-10 group than in the Tasi-1 group (*p* < 0.05) (Fig. 4).

## Discussion

In this study, we investigated the effects of Tasi, a Mel agonist, on oxidative stress, inflammation, apoptosis, and necrosis in TBI. As indicated by the reductions in TOS and the OSI, Tasi reduced oxidative stress and decreased the levels of inflammatory parameters such as TNF-α; necrosis parameters, such as RIPK1, RIPK3, and MLKL; and apoptosis parameters, such as Cas-3.

The release of cytokines in brain tissue in response to TBI activates many cellular mechanisms and causes damage to progress [12]. In addition to the development of various neurological complications depending on the area of the brain affected by damage, peripheral organ dysfunction may also occur due to increased permeability of the BBB [13]. To suppress all these troublesome processes, the number of drugs used routinely in TBI is very limited or provides inadequate therapeutic efficacy. Current studies are investigating the protective properties of drugs that are routinely used for tissue and cognitive functions in addition to their primary purposes, and the effects of new therapeutic agents are also being investigated [14].

The first mechanism of damage that develops in response to TBI is inflammation, and neutrophilic leukocytes are the first cells to migrate to the site of the lesion. The cytokines secreted by these leukocytes to battle infectious agents or foreign particles can also affect healthy tissues if they are secreted in excessive amounts. For example, TNF-α, an acute phase reactant, is known to be elevated in tissues following trauma, triggering other damage mechanisms. These mechanisms can activate each other and cause damage to become aggressive in a short period [15]. In this study, immunohistochemical TNF-α expression was detected in the trauma group, and meningeal hemorrhage and hyperemia detected by histopathological analysis supported the aforementioned inflammatory response. Compared with the Tasi-1 group, the Tasi-10 group suppressed these inflammatory responses secondary to trauma better and may have a greater neuroprotective effect in a dose-dependent manner. The activation of endogenously synthesized anti-inflammatory interleukins may play a role in reducing this normally observed inflammatory response. To investigate whether this response develops, new studies should be conducted in which a trauma model is created and Tasi treatment is administered for different durations and at different doses.

The inflammatory response is also known to induce oxidative stress. Oxidative stress activates the endogenous antioxidant enzyme system, which may be insufficient depending on the extent of brain damage. Obviously, increased ROS deepen the damage and trigger apoptosis or necrosis [16]. Hu et al. demonstrated for the first time that hydrogenated saline induces the modulation of necroptosis and neuroinflammation via the ROS/HO-1 pathway and provided a new perspective on the mechanisms underlying the neuroprotection and inhibition of inflammation and necroptosis by hydrogenated saline, as well as the evaluation of biological effects [2]. Feng et al. reported that atorvastatin prevented endoplasmic reticulum stress-mediated apoptosis in TBI mice through the NRF-2/HO-1 pathway [17]. In this study, increased levels of TOS and OSI, which are indicators of oxidative stress, in the trauma group indicate the development of oxidative stress secondary to trauma. On the other hand, the decrease in TAS capacity, which is an indicator of antioxidant activity, and NRF-2 and HO-1 gene expression in the trauma groups indicate that the antioxidant system is insufficient to balance the damage. At first glance, the decrease in TOS and OSI levels with Tasi treatment suggests that the compounds may be antioxidants. However, the fact that the TAS levels and NRF-2 and HO-1 gene expression levels in the treatment groups were close to those in the control group should be interpreted as preventing the expected decrease. To explain this situation, examining the same indicators of antioxidant activity in applications performed by forming groups in which only Tasi was given without trauma is necessary. This situation may also possibly be explained by the decreased oxidative stress response in parallel with the decreased inflammatory response with Tasi treatment.

Inflammation has also been shown to induce necrosis and apoptosis, particularly through cytokines such as TNF-α or IFN-γ [18]. For example, TNF-α is known to bind to its own receptors on the cell surface and induce apoptosis through caspase-8-mediated Cas-3 activation and necrosis by increasing RIPK1, RIPK3, and MLKL gene expression [19]. The association of TNF-α and Cas-3 detected by immunostaining supports this finding. As mentioned above, since Tasi reduces inflammation, apoptosis is likely reversed with a decreased TNF-α response.

TNF-α is one of the factors responsible for necrosis secondary to inflammation from various causes [20]. The activation of RIPK1, RIPK3, and MLKL, which are specific genes that are activated at the cellular level, can increase calcium entry into the cell by opening channels on the cell surface, and increased membrane permeability can cause leakage of the cell contents and necrosis [21]. Wehn et al. studied necroptosis, a form of programmed cell death mediated by the interaction of RIPK1 and RIPK3 that causes chronic brain injury after TBI, and showed that cognitive decline after TBI is dependent on RIPK1/RIPK3 expression in neurons [3]. In the genetic analysis of this study, the RIPK1, RIPK3, and MLKL expression levels detected in the trauma groups indicate that the development of necrosis is triggered in brain tissue. The decrease in the expression of these genes with Tasi treatment suggests that the drug may regress necrotic conditions secondary to inflammation. Necroptosis and oxidative stress are thought to affect each other indirectly. Although the direct signaling pathways linking them have not been examined, the damage caused by one mechanism is thought to trigger the other, and these processes may positively affect each other if they are treated.

Although this study provides important findings for understanding the potential protective effects of Tasi against traumatic brain injury (TBI), it has several limitations. First, the study was conducted only in an animal model, limiting the direct validity of the findings in humans. Therefore, clinical trials are needed to assess whether Tasi has similar effects on TBI in humans. Second, the dosages and treatment durations used in the study were limited, and more comprehensive studies are needed to examine the effects of different dosages and treatment durations. Furthermore, only the short-term effects of Tasi were examined, and long-term effects and potential side effects were not evaluated. A comparison of the effects of Tasi with those of other neuroprotective drugs has not been conducted, which constitutes a shortcoming in terms of assessing treatment efficacy. In addition, the inability to examine mRNA expression at the protein level and the inability to diversify with more detailed markers limit this study. Considering these limitations, future studies should be designed with a more comprehensive approach.

In conclusion, we observed that Tasi protected against brain injury through the NRF-2/HO-1 and RIPK1/RIPK3/MLKL pathways in rats with TBI.

## Data Availability

The data that support the findings of this study are not openly available due to reasons of sensitivity and are available from the corresponding author upon reasonable request.

## Abbreviations

TBI: Traumatic brain injuries
ROS: Reactive oxygen species
CNS: Central nervous system
TNF-α: Tumor necrosis factor-alpha
IFN-γ: Interferon gamma
BBB: Blood–brain barrier
Cas-3: Caspase-3
NRF-2: Nuclear factor erythroid 2-related factor 2
HO-1: Heme oxygenase-1
RIPK1: Receptor-interacting protein kinase-1
MLKL: Mixed lineage kinase domain-like
RIPK3: Receptor-interacting protein kinase 3
Mel: Melatonin
Tasi: Tasimelteon
SF: Saline
IP: Intraperitoneally
TAS: Total antioxidant status
TOS: Total oxidant status
OSI: Oxidative stress index
HE: Hematoxylin and eosin
