# Correction: Effects of Tasimelteon Treatment on Traumatic Brain Injury Through NRF-2/HO-1 and RIPK1/RIPK3/MLKL Pathways in Rats

**DOI:** 10.1007/s12035-025-05269-7

**Published:** 2025-08-14

**Authors:** Eyyüp Sabri Özden, Mustafa Soner Özcan, Mehtap Savran, Ilter Ilhan, Muhammet Yusuf Tepebası, Mehmet Abdulkadir Sevuk, Özlem Özmen

**Affiliations:** 1 Department of Anesthesiology and Reanimation, Faculty of Medicine, Suleyman Demirel University, Cunur, 32260 Isparta, Turkey; 2 Department of Pharmacology, Faculty of Medicine, Suleyman Demirel University, Isparta, Turkey; 3 Department of Biochemistry, Faculty of Medicine, Suleyman Demirel University, Isparta, Turkey; 4 Department of Genetic, Faculty of Medicine, Suleyman Demirel University, Isparta, Turkey; 5 Department of Pathology, Faculty of Veterinary Medicine, Burdur Mehmet Akif Ersoy University, Burdur, Turkey

**Correction: Molecular Neurobiology**

10.1007/s12035-025-04711-0

The original version of this article unfortunately contained errors in Materials and Methods section.

In this section, the authors described the trauma model in experimental animals, stating incorrectly that they used a "50 mg" ball. The corrected text should be "by dropping a 50 g ball from a height of 80 cm [7]." instead of "with a 50-mg ball from a height of 80 cm".

They also need to remove the sentences "Head trauma was induced according to the impact acceleration model of Cıkrıklar et al." and "The aim was to produce a trauma of 0.2 N according to Newton's law [7]." as this value changes when the weight is 50 g.

The original article has been corrected.

